# The Stability and Continuity of Maternally Reported and Observed Child Eating Behaviours and Feeding Practices across Early Childhood

**DOI:** 10.3390/ijerph15051017

**Published:** 2018-05-18

**Authors:** Faye Powell, Claire Farrow, Caroline Meyer, Emma Haycraft

**Affiliations:** 1 School of Psychology, Faculty of Creative Arts, Technologies & Science (CATS), University of Bedfordshire, University Square, Luton LU1 3JU, UK; 2 Department of Psychology, School of Life & Health Sciences, Aston University, Aston Triangle, Birmingham B4 7ET, UK; c.farrow@aston.ac.uk; 3 WMG, International Manufacturing Centre, University of Warwick and Warwick Medical School, Coventry CV4 7AL, UK; c.meyer@warwick.ac.uk; 4 School of Sport, Exercise & Health Sciences, Loughborough University, Loughborough LE11 3TU, UK; e.haycraft@lboro.ac.uk

**Keywords:** fussy eating, parental feeding practices, eating behaviour, child health, longitudinal research, observation, validation

## Abstract

Given that many eating behaviours and food preferences develop early in childhood and track across childhood, adolescence and into adulthood, interest has grown in the developmental trajectory of these behaviours. The aims of this study were twofold. First, to explore whether maternal reports of child eating behaviour and feeding practices are validated by independent observations of these constructs. Second, to explore the continuity and stability of both maternally reported and independently observed child eating behaviours and maternal feeding practices during early childhood. Sixty-five mothers completed measures of their child’s eating behaviour and their own feeding practices and mother–child dyads were observed during a family mealtime at approximately 3 and 4 years of age. Maternal reports of their child’s eating behaviours were validated by independent observations, however maternally reported feeding practices were not validated by observations of these behaviours. Maternally reported and independently observed child eating behaviours and parental feeding practices remained stable and showed continuity between 3 and 4 years of age, with the exception of child difficulty to feed and maternal pressure to eat which both significantly decreased over time. Findings provide an insight into the validity of maternal reports of fussy eating behaviour and parental feeding practices and the developmental trajectory of these behaviours across early childhood.

## 1. Introduction

Many children do not consume healthy and varied diets [1] and food fussiness and feeding problems are a common concern for parents and practitioners alike [2,3,4]. Fussy eating can present a barrier to healthy eating and a healthy BMI; associated problems include low fruit and vegetable intake [5,6] and essential nutrient deficiency [7]. Early childhood is a key period for the development of eating behaviour and food preferences [8], which can predict later eating attitudes, food preferences, food intake and BMI [9,10,11,12]. Therefore, a focus on early childhood and the early developmental trajectory of eating behaviours could provide an insight into how we can promote healthier eating and weight into adulthood. In order to appropriately explore developmental trajectory over time, it is important to consider both continuity within a group and stability in individuals; two theoretically and statistically independent concepts [13,14]. Continuity reflects consistency in mean group levels of behaviour over time, whereas stability reflects consistency in individual ranks of behaviour in a group over time [14]. Evaluating both concepts allows insight into the general course of development of a behaviour, as well as individual variation in that behaviour over time [15].

A small number of studies have explored the developmental trajectory of eating behaviours across childhood, e.g., [16,17,18]. Research using parent-report psychometric measures has found significant correlations in eating behaviour over time in children aged 2–4 [16] 2 to 5 [17] and 4 to 11 [18], suggesting that parents’ descriptions of their child’s fussy eating behaviours may show individual stability across childhood. However, findings suggest that continuity in mean group levels may differ across childhood. For example, a small-scale study reported consistency in mean levels of food avoidant eating behaviours across early childhood (between 2–5 years of age) [17] whereas findings in 4–11 year olds suggest there may be developmental increases in children’s obesogenic eating behaviours during this time [18]. Although these studies provide a preliminary insight into the developmental trajectory of eating behaviours across childhood [16,17,18], they have relied on parental reports of these behaviours, which may be subject to bias. While some studies have found that mothers are quite accurate in their reports of mealtime interactions [19,20,21], other studies have found that independent observations do not validate maternal reports [22] or that the accuracy of maternal reports is dependent on child weight [15]. Therefore, the use of observational methods to explore the developmental trajectory of eating behaviour in early childhood would strengthen and extend current findings within this field.

Although some elements of children’s eating behaviour, such as appetite, may reflect intrinsic characteristics of the child [23], eating behaviours can also develop as a result of a child’s interactions with the environment; part of which involves caregivers or parents [24]. Parental feeding practices represent a contributory environmental factor in the development, persistence and prevention of fussy eating and feeding problems [25,26]. Yet despite this, only a small amount of research has explored whether parental feeding practices are also stable and continuous over time. The limited research in this field has predominantly focused on controlling feeding practices (e.g., pressure to eat, restriction of food, and monitoring) and studies have indicated that such strategies are stable from 1 to 2 years of age [27], from 2 to 5 years of age [15,16], and between 5 and 7 years of age [28]. With regard to continuity in parental feeding practices, only one study to date has explored this in parents of children aged 2–5 years old; results demonstrated continuity in mean levels of parental restriction and monitoring over time whereas levels of maternal pressure to eat were found to significantly increase over time [17]. Within older samples, parents report allowing their children more independence across middle childhood, between 4, 7 and 9 years [29] with decreases in their use of pressure to eat, monitoring and restriction between 7 and 10 years of age [30].

While the preceding studies provide an insight into the continuity and stability of controlling feeding practices, no research to date has explored the early trajectory of other measurable maternal feeding practices such as involvement in food preparation or encouraging a balanced and varied intake. These potentially healthful practices have been previously associated with more adaptive eating behaviour in children such as less food fussiness and higher intake of fruits and vegetables [31,32,33]. Importantly, such feeding practices are potentially modifiable and could be targeted for inventions to improve child dietary intake, eating behaviour and weight status across childhood. The development of broader measures of parental feeding practices such as the Comprehensive Feeding Practices Questionnaire (CFPQ) [34] increase the scope of research using parental report, however observational measures allow further exploration of subtle behaviours and interactions between mother–child dyads during mealtimes that caregiver might not be aware of. These include constructs such as maternal sensitivity and interactional conflict during mealtimes, which have also been implicated in the development of child eating behaviours [35], but are more difficult to assess using questionnaire methods. Therefore, the current study utilises observations of maternal–child interactions during family mealtimes to both validate maternal-reports of child eating behaviour and maternal feeding practices and to further explore the continuity and stability of these behaviours.

The first aim of the present study was to explore whether maternal reports of child eating behaviour and feeding practices are validated by independent observations of these behaviours in children aged 2–4 years old. Secondly, this study aimed to explore the continuity and stability of both maternally reported and independently observed child eating behaviours and maternal feeding practices during early childhood. It was hypothesised that children’s eating behaviours would be significantly correlated over time, showing stability between 3 and 4 years of age and that there would be continuity in mean group levels of eating behaviour over time. It was also hypothesised that feeding practices would be significantly correlated over time, showing individual stability between 3 and 4 years of age. However, due to previously inconsistent findings in terms of continuity in mean group levels, it was hypothesised that these behaviours may change between 3 and 4 years of age.

## 2. Materials and Methods

### 2.1. Participants

Seventy-one mothers of children aged between 2–4 years were recruited through nurseries, pre-schools and children’s centres in the East Midlands and South East region of the UK. At time point 1 (TP 1), these participants completed a set of standardised questionnaires about them and their child and were observed with their child during a typical mealtime in their home (TP 1; mean child age 3.54 years; range = 2.00–4.83, SD = 1.00). Sixty-five of these dyads were followed up 12 months later (Time point 2: TP 2) and completed the same set of questionnaires and another home mealtime observation (TP 2; mean child age 4.40 years; range = 3.10–5.62, SD = 1.13). Mann–Whitney U tests demonstrated that there were no significant differences between participants who dropped out of the study after baseline assessment (TP 1) and those who took part in the follow-up study (TP 2) in relation to their demographic background or maternal reports and independent observations of their child’s eating behaviour and their own feeding practices at 3 years of age (*p* > 0.05).

Mothers were on average 35.94 years old (range = 27.42–46.92, SD = 4.19) at TP 1 and there were 34 mothers of boys and 35 mothers of girls. At the 12 month follow up (TP 2), mothers were on average 37.03 years old (range = 28.42–48.12, SD = 4.25) and there were 32 mothers of boys and 33 mothers of girls. Mothers had on average 5.02 years of education post-16 (range = 1.00–9.00 years, SD = 1.76) and 97% of the mothers in this sample reported that they were White British. Using the Standard Occupational Classification 2000 [36], participants reported a range of occupations from 1 (managers and senior officials) to 8 (elementary occupations), with a modal occupation level of 4 (administrative and secretarial occupations). Annual family income ranged from <£15,000 to >£75,000 with a modal income of £45,000–£60,000.

### 2.2. Measures and Procedure

Following ethical approval from Loughborough University Ethical Advisory Committee (ethics code: G10-P9), recruitment and informed consent, standardised questionnaire packs were completed by mothers, approximately 12 months apart (range 11.10–12.90 months) at TP 1 and TP 2. Mother–child dyads were also observed (and recorded using a video camera) during a typical lunch or evening meal at the family’s home at TP 1 and TP 2. Mothers received questionnaire packs via post to complete prior to the mealtime visit. These were collected at the home visit.

Mothers were asked to prepare a typical meal and to feed their child and conduct the mealtime as they usually would. The video camera was set up and the researcher waited in a different room during the mealtime. Following the meal, the mother was asked to rate how typical the mealtime was on a scale from “1” (very untypical meal/ behaviour) to “5” (very typical meal/behaviour). Exclusion from the study was planned if any parents gave a score of less than 3, but this was not necessary. Where consent was provided from parents and children, objective measures of height and weight were collected by the researcher using a Child Growth Foundation’s Leicester height measure and digital Secca scales, measured to the nearest 0.1 centimetre and 0.1 kilogram, respectively. Mothers provided background demographic information and completed two self-report measures at TP 1 and TP2.

*Children’s Eating Behaviour Questionnaire (CEBQ)* [37]. Parents completed four subscales of the CEBQ: Food fussiness which measures pickiness with regard to the type of food the child is willing to eat; Slowness in eating which assesses the pace at which the child consumes their food; Satiety responsiveness which evaluates a child’s fullness threshold; and, Enjoyment of food which measures the child’s interest and enjoyment in food and eating. Mothers rated the frequency that their child exhibited a range of behaviours using a 5-point Likert scale ranging from never (1) to always (5); higher scores indicated a greater prevalence of that behaviour. The CEBQ demonstrates good psychometric properties, with reports of good test–retest reliability (r = 0.52–0.87), stability over time and internal validity, with Cronbach’s α for the subscales ranging from 0.72 to 0.91 [37,38]. The subscales have also been found to correlate well with behavioural measures of these constructs [38]. Reliability analysis within this sample at TP 1 and TP 2 demonstrated good Cronbach’s *α* for the subscales, which ranged from 0.77–0.89.

*Comprehensive Feeding Practices Questionnaire (CFPQ)* [34]. Mothers completed nine subscales of the CFPQ categorised into three areas: Control (pressure to eat, restriction of food for health, restriction of food for weight control, monitoring); Use of Food for Behaviour Regulation (using food to regulate child emotional states and using food as a reward); and, Environment (encouraging balanced and varied food intake, providing a healthy environment, involving child in food planning and preparation). Responses were provided on a 5-point Likert scale ranging from never to always or disagree to agree; higher scores indicated a higher prevalence of that feeding practice. The CFPQ has demonstrated good internal reliability for its 12 subscales with a mean of 0.73 (range = 0.58–0.87) and good convergent and discriminant validity [39]. Reliability analysis within this sample indicated good reliability with Cronbach’s *α* ranging from 0.65–0.79.

*Mealtime Observation*. In order to explore the validity of maternal reports of child eating behaviour and maternal feeding practices, aspects from various observational assessment measures were used to assess these constructs.

*Child Eating Behaviour*. The Child Mealtime Coding System (CMCS) [40] and an adapted scale from the Behavioural Coding Inventory (BCI) [41] were used to gain independent observations of child speed of eating, food refusals, how difficult the child was to feed, and positive and negative vocalisations about food.

*Maternal feeding practices and mealtime behaviour*. The Family Mealtime Coding System (FMCS) [22] was used to measure parental control during the meal. Over the course of the meal, a count was made for each time the mother pressured their child to consume more food, physically prompted the child to consume more food, and used incentives such as conditions or rewards to get their child to eat. Two subscales from the Feeding Interaction Scale (FIS) [42] were used to rate interactional conflict between mother–child dyads during the meal and maternal feeding sensitivity. This was rated on a 9-point Likert scale with higher scores reflecting a higher occurrence.

*Inter-rater reliability*. To determine inter-rater reliability, 20% of the observations (selected at random) were coded by a s second independent trained observer. Mean inter-rater reliability across TP 1 and TP 2 was 0.84 (range 0.79–0.94; intra-class correlation co-efficient) and the mean level of significance was *p* < 0.001, demonstrating good reliability.

### 2.3. Data Analysis

Preliminary analysis of the data using Shapiro–Wilk tests demonstrated that data were predominantly non-normally distributed and so non-parametric statistics were used. After conducting descriptive statistics, a series of two-tailed Spearman’s rho correlations were completed to explore whether maternal reports of child eating behaviour and maternal reports of their feeding practices were validated by independent observations of these constructs. Following this, difference scores were calculated between 3 and 4 years of age by subtracting scores at age 3 from scores at age 4. This allowed mean change scores to be calculated, with positive values representing an increase in the variable over time, and negative values representing a decrease. Spearman’s two-tailed correlations showed that maternal age, occupation, income and child age were not significantly correlated with the degree of change in maternal feeding practices or child eating behaviours. In addition, Mann–Whitney U tests showed that there were no significant differences between male and female children in the degree of change on these variables between age 3 and 4. Therefore, these demographic variables were not included within any of the further analyses. Mann–Whitney U tests also indicated that there were no significant differences dependent on whether or not the father was present at the mealtime (*n =* 19 at TP 1; *n =* 18 at TP 2).

Cote and Bornstein’s (2003) paradigm, adjusted to account for non-normal data, was used to evaluate the stability and continuity of the variables [14]. Two-tailed Spearman’s rho correlations were used to explore the stability of the variables and Wilcoxon signed rank tests were used to explore continuity between 3 and 4 years of age. Where variables did not show continuity (demonstrated by significant differences on Wilcoxon matched pairs test), effect sizes (ES) were calculated. This was achieved by dividing the mean change score for the variable by the standard deviation (SD) of the mean score for the variable at the first time point [43].

## 3. Results

### 3.1. Characteristics of the Sample

At time point one (TP 1), mothers’ mean reported BMI was 23.02 (SD = 3.23, *N* = 56), suggesting that they were on average of healthy weight (BMI < 25 [44]). Twenty-three percent of the sample reported a BMI that would be considered as overweight (BMI ≥ 25) or obese (BMI ≥ 30) [45], which is similar to national averages within the UK [45]. Objective measures of height and weight were also obtained for 29 mothers. Interclass correlations between self-reports and objective measures had a coefficient of 0.73, indicating that mothers were broadly accurate in reporting their height and weight.

Children’s height and weight were reported by fifty-four mothers. The mean maternally reported BMI *Z*-score [46] was −0.02 (range = −3.24–2.87, SD = 0.91), suggesting that the sample of children, on average, had a healthy BMI. Objective measures of height and weight were also obtained for 60 of the children (15 parents or their child did not consent). The mean BMI *Z*-score (based on objective measures of height and weight) was 0.62 (Range = −2.62–2.87, SD = 0.89), which while higher than self-reported BMI, similarly suggests a healthy BMI; 86.7% of the sample were healthy weight, 5% were underweight and 8.3% were overweight or obese [47]. These rates of overweight are slightly lower than national averages for children in this age range within the UK [45]. Interclass correlations between maternal reports and objective measures of child BMI had a coefficient of 0.48, indicating that mothers were only moderately accurate in reporting their child’s height and weight.

Descriptive statistics for maternal report and observed child eating behaviours are presented in Table 1 and for maternal feeding practices in Table 2. Mean scores for the CEBQ, CMCS, BCI, CFPQ, FMCS and FIS are similar to other published data in comparable samples [22,34,37,40].

### 3.2. Validity of Maternal Reports of Child Food Avoidant Eating Behaviour

Two-tailed Spearman’s correlations were used to explore whether maternal reports of child eating behaviour were validated by independent observations. As indicated in Table 3, maternal reports of children’s eating behaviour were significantly correlated with a number of independent observations. Higher levels of maternally reported food fussiness and lower levels of maternally reported food enjoyment were associated with independent observations of more frequent food refusal, a slower eating speed, fewer positive comments about food and the child being rated as more difficult to feed. Lower reported food enjoyment was also correlated with observations of a higher frequency of negative comments about food. Children who were reported to eat more slowly were observed to eat more slowly, refused more mouthfuls of food and made more negative vocalisations about food. Children with higher levels of maternally reported satiety responsiveness were observed to eat more slowly and made fewer positive comments and more negative comments about food.

### 3.3. Validity of Maternal Reports of Feeding Practices

To explore whether maternal reports of their own feeding practices would be validated by independent observations, further two-tailed Spearman’s correlations were employed. As indicated in Table 4, maternally reported feeding practices were not significantly correlated with independent observations of their feeding practices.

### 3.4. Continuity of Child Eating Behaviour and Parental Feeding Practices between 3 and 4 Years of Age

Table 1 and Table 2 present the mean change scores for maternally reported and observed variables between 3 and 4 years of age and Wilcoxon matched signed rank tests to explore continuity. Table 1 demonstrates significant continuity in all maternal reported and most observed child eating behaviours between 3 and 4 years of age, with the exception of child difficulty to feed which significantly decreased between 3 and 4 years. Table 2 indicates there was also continuity in all maternal reported and most observed maternal feeding practices and behaviours, with the exception of maternal pressure to eat which significantly decreased between 3 and 4 years. The effect size (ES) of these changes show relatively small decreases in pressure to eat (ES = 0.28) and child difficulty to feed (ES = 0.30) over time.

### 3.5. Stability of Child Eating Behaviours and Parental Feeding Practices and Behaviours between 3 and 4 Years of Age

Table 5 shows the correlations over time for maternal reports and independent observations of child eating behaviour at TP 1 and TP 2. All variables were significantly, positively correlated over time, indicating stability in these measures of eating behaviour; correlations ranged from 0.43 (positive comments about food) to 0.81 (food fussiness).

Table 6 shows the correlations over time for maternal report and independent observations of parental feeding practices and behaviours at TP 1 and TP 2. All maternal feeding practices and behaviours were significantly correlated over time suggesting stability in these variables in children of this age; significant correlations ranged from 0.48 (balance and variety) to 0.75 (restriction for weight).

## 4. Discussion

The first aim of this study was to evaluate whether maternal reports of child eating behaviour and feeding practices were validated by independent observations of these behaviours. Maternal reports of their child’s eating behaviour correlated with independent observations, however, contrary to predictions, maternally reported feeding practices did not correlate with independent observations of these behaviours. The second aim of the study was to explore the continuity and stability of both maternally reported and independently observed child eating behaviours and maternal feeding practices during early childhood. The results demonstrated stability in individual ranks of maternally reported and observed child eating behaviours and maternal feeding practices between 3 and 4 years. The study also found continuity in mean group levels of both maternal reports and independent observations of children’s eating behaviours and maternal feeding practices over time, with the exception of child difficulty to feed and maternal pressure to eat, both of which significantly decreased between 3 and 4 years age.

Although self-reports can be subject to bias, the results of the present study support previous work which has found that mothers are reasonably accurate in reporting their children’s eating behaviour [19,20,48,49]. This suggests that mothers are in tune with their child’s eating behaviour, possibly because mothers are often the primary caregiver and are therefore likely to engage in daily interactions with their children around food, particularly in this younger age group. However, the poor correspondence between observed and self-reported feeding practices highlights the importance of utilising observational measures, as mothers’ reports of their own feeding practices may not be an accurate reflection of their actual behaviour. It is possible that mothers may underestimate their use of coercive strategies when completing self-report measures. However, it should also be acknowledged that observational measures may lead mothers to act differently because they are being observed. It is important to note that this study only explored the validity of maternal reports of pressure to eat and the use of rewards. Future research should seek to find methods to measure and observe a wider range of feeding practices and mealtime behaviours and those that may expand beyond the family mealtime. For, example, the use of restriction, which may be more likely to occur during snack times, or when unhealthy foods are offered to or requested by the child, rather than during a meal, where the foods offered are likely to have been chosen by the parent.

Supporting the hypotheses and extending previous findings [16,17,18], our results suggest that both maternal reports and independent observations of children’s eating behaviours are stable across early childhood. Significant correlations were found, suggesting, for example, that children with high levels of food fussiness or food refusal at 3 years of age still had relatively high scores on these variables at 4 years of age. This supports evidence that eating behaviours develop early in childhood and may persist over time [8,17,18], perhaps not dissimilarly to the stability of other traits such as child temperament [50]. These findings highlight a need for future longitudinal research to explore these behaviours earlier in childhood, following children from birth through weaning and early childhood to provide further insight into the origin, nature and projection of eating behaviour.

The findings of this study also indicate continuity in mean group levels of maternally reported and observed feeding practices between 3 and 4 years of age. This supports previous evidence that reported continuity in parental reports of food fussiness, slowness in eating, satiety responsiveness and enjoyment in food over time in children aged 2–5 years old [17]. However, these findings are contrary to those with older children aged 4–11 years old, where satiety responsiveness, slowness in eating and food fussiness were found to significantly decrease over time, while enjoyment of food significantly increased [18]. This suggests that while children’s eating behaviours may be relatively stable during early childhood, changes may occur later as children progress into middle childhood. This is perhaps not unexpected given that factors such as food neophobia have been found to peak between 2 and 6 years of age [51] and then decline across childhood [52].

This is the first study to date to explore continuity in observed child eating behaviour, and while the results are largely parallel to maternal report, there was one exception: mean levels of observed child difficulty to feed reduced over time. This may reflect developmental changes as children become more autonomous, independent eaters or it could highlight a discrepancy in measurements. As the incidence of feeding problems or eating issues is often elevated when reported by caregivers compared to healthcare professionals [53], independent observations may provide a more accurate indication of eating behaviours than self-report measures. It is essential that future research continues to utilise observational measures to validate maternal reports so that the early trajectory of child eating behaviour can be better understood.

As parental feeding practices may influence children’s emerging eating behaviours, an important aim of this study was to explore the continuity and stability of these factors. Building on previous work, this study was the first to explore the trajectory of a broader range of maternal feeding practices such as using food as a reward, feeding for emotion regulation and potentially adaptive behaviours such as involvement and encouraging varied food intake. Supporting previous findings investigating the stability of maternal controlling feeding practices [17,27,28], all of the measured maternal feeding practices and behaviours within this study demonstrated good stability and were significantly correlated over time. These findings add to the reliability of the CFPQ [34] as a measure of parental feeding practices over time.

Despite mixed and often equivocal evidence from previous studies [17,30], our study found continuity in mean group levels over time for all maternal feeding practices, with the exception of observed maternal pressure to get the child to eat which was found to significantly decrease over time. This supports findings from Webber et al. [30] in children aged 7–10, but contradicts findings in 2–5 year old children, where parents’ use of pressure to eat was found to increase over time [17]. However, the present findings were incongruent, with maternal reports of pressure to eat demonstrating stability. Again, this could reflect issues with the validity of maternal report and further studies using observational measures are needed to provide clarity. Observed child difficulty to feed and maternal pressure to eat were the only variables found to reduce over time. Evidence has suggested that parental feeding practices may be employed in response to a child’s eating behaviour [28,54,55], therefore it is possible that as children become more autonomous and less difficult at mealtimes, parents feel less need to pressure them to eat. However, there is also evidence of a causal link between pressure to eat and feeding problems [55] therefore inferences about the direction of this relationship should be made cautiously.

The findings of this research build upon previous knowledge concerning the stability and continuity of eating behaviours and feeding practices during early childhood. It is the first study, to date, to explore the developmental trajectory of child eating behaviours and parents’ feeding practices using both maternal-report and observational measures. It also incorporates a broader range of measurable parental feeding practices and behaviours associated with child eating behaviour than previous work. Importantly, this study has not focused solely on controlling feeding practices and it has included potentially healthful practices that have been previously associated with more adaptive eating behaviour in children [31,32,33]. Although it is acknowledged that the small, fairly homogenous sample may limit the generalisability of the findings, studies incorporating both observational measures and longitudinal designs are rare and often use smaller samples. Replication of the study within a more diverse sample would strengthen conclusions and allow the findings to be generalised to other socio-demographic groups. The findings of this study also relate to mothers only. The mealtime observation was designed to be as ‘typical’ as possible with no stipulations about who should be present, the circumstances of the meal or the food that was prepared. Mothers were asked to prepare a typical meal and to feed their child and conduct the mealtime as they usually would and fathers were present in approximately 25% of the mealtimes. Preliminary data analysis identified no significant differences in eating behaviour or maternal feeding practices according to whether the child’s father was present; therefore, paternal influence was not explored further. However, due the small number of fathers present, the data may be underpowered to detect significant differences according to paternal presence. Future studies may benefit from purposefully recruiting fathers and exploring the role of fathers during mealtime interactions in greater depth. Another important consideration when interpreting these findings is the age range of the children, who were recruited aged between 2–4 years old and followed up 12 months later at 3–5 years old. One might expect there to be within-group variability in eating behaviour and feeding practices dependent on child age, affecting the validity of measures of continuity and stability amongst this sample. However, data screening revealed that child age was not significantly correlated with the degree of change in maternally reported or independently observed child eating behaviour or maternal feeding practices, suggesting that changes did not significantly differ depending on child age.

## 5. Conclusions

In summary, the findings of this study suggest that while mothers may be able to accurately report their children’s eating behaviour, self-reports of their own feeding practices are less accurate. Mothers’ reports and independent observations of both child eating behaviour and maternal feeding practices remained predominantly stable and continuous between 3 and 4 years of age. Through the use of observational measures, the results provide novel evidence that validated reports of child eating behaviour are stable and continuous across early childhood. Given that this period in childhood has been identified as a critical time in the development eating behaviours and food preferences, these findings highlight a need to continue to explore the developmental trajectory of eating behaviour from as early in life as possible; following eating behaviour from infancy and weaning through the first few years of life. In addition, given findings from Ashcroft et al. [18], future studies should seek to continue to follow children later into childhood, to ascertain whether fussy eating behaviours, while apparently stable and continuous across early childhood, do in fact decrease in middle childhood. Importantly, this study provides evidence of the stability and continuity of maternal feeding practices and mealtime behaviours that have not been previously explored. A better understanding of the trajectory of parental feeding practices and their influence on children’s emerging eating behaviours would have beneficial implications for future treatment and prevention of feeding problems and food fussiness in children.

## Figures and Tables

**Table 1:** Descriptive statistics and Wilcoxon signed rank tests for maternally reported and observed child eating behaviour over time.

Child Eating Behaviour	TP 1 (3 Years) Mean (SD)	TP 2 (4 Years) Mean (SD)	Mean Change TP1–TP 2 (SD)	Wilcoxon *Z*-Score
Maternal Report (CEBQ)				
Food fussiness	2.89 (0.92)	2.88 (0.92)	−0.01	−0.07
Slowness in eating	3.00 (0.79)	2.91 (0.81)	−0.09	−1.40
Satiety responsiveness	3.18 (0.67)	3.09 (0.70)	−0.09	−1.76
Enjoyment of food	3.68 (0.73)	3.69 (0.88)	0.01	0.03
Observed (BCI; CMCS)				
Speed (mouthfuls per minute)	3.14 (1.57)	3.07 (1.32)	−0.07 (1.91)	−0.20
Food refusals ^c^	4.50 (5.94)	4.12 (7.18)	−0.38 (3.64)	−0.74
Difficult to feed ^r^	2.39 (1.08)	2.08 (0.83)	−0.31 (0.93)	−2.67 *
Positive vocalisations about food ^c^	3.57 (3.34)	3.15 (2.90)	−0.42 (3.70)	−0.92
Negative vocalisations about food ^c^	1.55 (2.95)	1.45(2.77)	−0.10 (2.12)	−0.28

* *p* < 0.05; CEBQ = Children’s Eating Behaviour Questionnaire; BCI = Behavioural Coding Inventory; CMCS = Child Mealtime Coding Scheme; c = counts (i.e., the frequency of occurrence across mealtime); r = ratings (i.e., objective rating (1–5) with a higher score reflect higher rating).

**Table 2:** Descriptive statistics and Wilcoxon signed rank tests for maternally reported and observed maternal feeding practices and behaviours over time.

*Maternal Faternal Fe 3−4 Years (SD) n Test Re-Test Reliability for the Observational Measures Used.s: From Pregnancy through the First Poeeding Practices and Behaviours*	TP 1 (3 Years) Mean (SD)	TP 2 (4 Years) Mean (SD)	Mean Change TP 1–TP 2 (SD)	Wilcoxon Z-Score
Maternal Report (CFPQ)				
Pressure	3.15 (0.85)	3.04 (0.89)	−0.11 (0.69)	−1.20
Restriction for weight	1.91 (0.65)	1.78 (0.59)	−0.13 (0.50)	−2.26
Restriction for health	3.19 (0.87)	3.21 (0.92)	0.02 (83)	0.47
Monitoring	4.21 (0.70)	4.28 (0.65)	0.05 (0.42)	1.04
Emotional regulation	1.91 (0.59)	1.85 (0.46)	−0.06 (0.49)	−1.15
Food as a reward	2.24 (0.78)	2.40 (0.76)	0.16 (0.74)	1.10
Balance and variety	4.27 (0.61)	4.37 (0.53)	0.10 (0.57)	0.88
Healthy environment	3.97 (0.79)	4.03 (0.80)	0.06 (0.65)	1.12
Involvement	3.54 (0.45)	3.62 (0.51)	0.08 (0.49)	0.92
Observed (FMCS; FIS)				
Pressure to eat ^c^	4.05 (5.03)	2.63 (4.24)	−1.42 (2.92)	−3.65 *
Physical prompts ^c^	5.14 (10.82)	3.60 (7.60)	−1.54 (8.46)	−1.25
Incentives and rewards ^c^	2.02 (3.08)	1.83 (3.05)	−0.19 (2.35)	−0.93
Feeding sensitivity ^r^	6.60 (1.64)	6.86 (1.86)	0.26 (1.44)	1.54
Interactional conflict ^r^	3.05 (1.82)	2.97 (2.16)	−0.08 (1.55)	−3.93

* *p* < 0.001; CFPQ = Comprehensive Feeding Practices Questionnaire; FMCS = Family Mealtime Coding System; FIS = Feeding Interaction Scale; ^c^ = counts (i.e., the frequency of occurrence across mealtime); ^r^ = ratings (i.e., objective rating (1–9) with a higher score reflect higher rating).

**Table 3:** Spearman’s rho correlations between maternal reports and independent observations of child eating behaviour.

		Maternal Report of Child Eating			
		Food Fussiness	Slowness in Eating	Satiety Responsiveness	Enjoyment of Food
Observed	Speed (mouthfuls per minute)	−0.34 *	−0.38 **	−0.31 *	0.38 **
	Food refusals	0.40 **	0.30 *	0.21	−0.45 **
	Difficult to feed	0.43 **	0.29	0.23	−0.45 **
	Positive vocalisations food	−0.44 **	−0.03	−0.31 *	0.32 *
	Negative vocalisations food	0.26	0.37 *	0.33 *	−0.43 **

* *p* < 0.01, ** *p* < 0.001 (two-tailed).

**Table 4:** Spearman’s rho correlations between maternal reports and independent observations of maternal feeding practices.

		Maternal Report	
		Pressure to Eat	Food as a Reward
Observed	Pressure	0.19	0.12
	Physical Prompt	0.06	−0.01
	Conditions and incentives	0.06	0.02

Nothing significant at *p* ≤ 0.05 (two-tailed).

**Table 5:** Spearman’s rho correlations for maternal reports and observed child eating behaviour between 3 and 4 years of age.

Child Eating Behaviour	rho Between TP 1 (3 Years) and TP 2 (4 Years)
Maternal report (CEBQ)	
Food fussiness	0.81 *
Slowness in eating	0.67 *
Satiety responsiveness	0.63 *
Enjoyment of food	0.73 *
Observed (BCI; CMCS)	
Speed (mouthfuls per minute)	0.68 *
Food refusals	0.65 *
Difficult to feed	0.56 *
Positive vocalisations about food	0.43 *
Negative vocalisations about food	0.46 *

* *p* < 0.001 (two-tailed); CEBQ = Children’s Eating Behaviour Questionnaire; BCI = Behavioural Coding Inventory; CMCS; Child Mealtime Coding Scheme.

**Table 6:** Spearman’s rho correlations for maternal report and observed maternal feeding practices and behaviours at 3 and 4 years of age.

Maternal Feeding Practices and Behaviours	rho Between TP 1 (3 Years) and TP 2 (4 Years)
Maternal Report (CFPQ)	
Pressure to eat	0.66 **
Restriction for weight	0.75 **
Restriction for health	0.58 *
Monitoring	0.72 **
Emotional regulation	0.62 **
Food as a reward	0.51 *
Balance and variety	0.48 **
Healthy environment	0.59 **
Involvement	0.52 **
Observed (FMCS; FIS)	
Pressure to eat	0.63 **
Physical prompts	0.57 **
Incentives and rewards	0.57 **
Feeding sensitivity	0.62 **
Interactional conflict	0.61 **

* *p* < 0.01, ** *p* < 0.001 (two-tailed); CFPQ = Comprehensive Feeding Practices Questionnaire; FMCS = Family Mealtime Coding System; FIS = Feeding Interaction Scale.
